# Feasibility of a point‐of‐care ultrasound protocol for cardiorespiratory evaluation of horses in different clinical settings

**DOI:** 10.1111/jvim.16674

**Published:** 2023-03-28

**Authors:** Kari E. Bevevino, Noah D. Cohen, Sonya G. Gordon, Cristobal Navas de Solis

**Affiliations:** ^1^ Department of Large Animal Clinical Sciences, College of Veterinary Medicine & Biomedical Sciences Texas A&M University College Station, Texas 77843 USA; ^2^ Department of Small Animal Clinical Sciences, College of Veterinary Medicine & Biomedical Sciences Texas A&M University College Station, Texas 77843 USA; ^3^ Department of Clinical Sciences, New Bolton Center University of Pennsylvania Kennett Square, Pennsylvania 19348 USA

**Keywords:** cardiac, equine, point of care, respiratory, sonogram, ultrasound

## Abstract

**Background:**

A point‐of‐care ultrasound (POCUS) protocol for evaluation of the cardiac and respiratory systems in horses does not exist.

**Objectives:**

(a) Describe the windows of a POCUS protocol for cardiorespiratory assessment of horses (CRASH); (b) Estimate the number of acoustic windows that can be acquired by a sonographer‐in‐training; (c) Estimate the time required to complete the protocol for specific groups of horses; (d) Describe the sonographic abnormalities detected in horses presented with cardiovascular, respiratory, or systemic disease.

**Animals:**

Twenty‐seven healthy horses, 14 horses competing in athletic events, and 120 horses with clinical disease.

**Method:**

A pocket‐sized ultrasound device was used to acquire 7 sonographic cardiorespiratory windows in various clinical scenarios. The duration of the examination was timed, and images were evaluated for diagnostic quality. Abnormalities in horses with clinical disease were determined by an expert sonographer.

**Results:**

The CRASH protocol could be performed in healthy and diseased horses in hospital, barn, and competition settings between 5.5 ± 0.9 (athletic horses) and 6.9 ± 1.9 min (horses with clinical disease). Thoracic windows were obtained most consistently, followed by right parasternal long‐axis echocardiographic windows. Frequently detected abnormalities were pleural fluid, lung consolidation, B‐lines, and moderate‐to‐severe left‐sided heart disease.

**Conclusions:**

The CRASH protocol was feasible using a pocket‐sized ultrasound device in various groups of horses, could be completed rapidly in a variety of settings, and frequently identified sonographic abnormalities when evaluated by an expert sonographer. The diagnostic accuracy, observer agreement, and utility of the CRASH protocol merit further evaluation.

**Abbreviations**
BCS: body condition score
CAD: caudodorsal thoracic
CAV: caudoventral thoracic
CPUS: clinician‐performed ultrasound
CRASH: cardiorespiratory assessment with sonography in the horse
L2C: 2 chamber long axis
LVOT: left ventricular outflow tract
POCUS: point of care ultrasound
R4C: 4 chamber long axis
SAAo: short axis at the level of the aorta
SAch: short axis at the level of the chordal attachments
SD: standard deviations

## 1 INTRODUCTION

Ultrasonography allows rapid, stall‐side, real‐time assessment of multiple body systems in horses. It is used frequently in specialty and general practice in hospital and field settings. Comprehensive ultrasonography and examination of complex disease often entails detailed evaluations that might require advanced training and can be time‐consuming. Point‐of‐care ultrasound (POCUS) or clinician‐performed ultrasound (CPUS)^1^ protocols are increasingly used in veterinary and human medicine because of technological and training advances. Focused ultrasonographic protocols are time‐sensitive, goal‐oriented assessments that answer focused clinical questions based on a patient's clinical signs.

Point‐of‐care ultrasound protocols for cardiac and lung evaluation first were introduced in human medicine in the 1990s. Since then, different standardized protocols that offer valuable information in specific clinical scenarios have emerged.^2^ Some of these protocols have become particularly relevant during the current coronavirus 2019 (COVID‐19) disease pandemic.^3^, ^4^ Multiple studies have evaluated the utility and accuracy of focused cardiac and lung ultrasound examinations as well as the training programs that have been designed to teach focused cardiac ultrasound examination in humans and small animals.^5^, ^6^, ^7^, ^8^, ^9^, ^10^, ^11^, ^12^, ^13^ Focused ultrasound examinations that combine evaluation of both the respiratory and cardiac systems exist for humans but none currently is described for small animals or horses.^14^ Common scenarios in which focused cardiac and respiratory ultrasound examination have been implemented for human and small animal medicine are emergency and critical care, resource‐limited settings, and primary care practice. In these instances, comprehensive ultrasound examination or other forms of advanced imaging may not be readily available or needed, and a focused examination can increase diagnostic accuracy of common cardiac and respiratory diseases.^14^ The American Society of Echocardiography and other medical organizations have published multiple consensus statements to establish definitions, protocols, recommendations, and limitations, and to review information regarding diagnostic accuracy of focused echocardiography and lung ultrasound examination. These guidelines often emphasize that focused ultrasound examinations are not intended to replace comprehensive echocardiograms, comprehensive sonograms, or the work of specialists but to augment diagnostic procedures and improve medical care.^15^, ^16^, ^17^

Point‐of‐care ultrasound protocols for the evaluation of adult horses with colic (FLASH) and high‐risk pregnancies have been evaluated.^18^, ^19^ Preliminary concepts of POCUS protocols for horses designed to define standards of training, indications and diagnostic accuracy of focused echocardiography, and POCUS for cardiorespiratory assessment recently were introduced.^20^ The goal of these protocols is to make medical care accessible while maintaining or improving standards of care in general and specialty practice.

Our goal was to describe a protocol for focused cardiorespiratory ultrasound (CardioRespiratory Assessment with Sonography in the Horse [CRASH]) targeted to detect cardiac and respiratory diseases in the horse. This protocol combines assessment of both cardiac and respiratory systems because diseases of either system can sometimes be difficult to differentiate clinically, especially in emergent or time‐sensitive scenarios. Our specific objectives study were: (a) to select and describe the windows of CRASH, a POCUS protocol for cardiorespiratory assessment of horses; (b) to determine the number of acoustic windows for CRASH that can be acquired by a sonographer in training in certain clinical scenarios; (c) to determine the time required to complete the CRASH protocol for specific groups of horses; and (d) to describe the abnormalities detected by an expert sonographer using the CRASH protocol in horses presented with cardiovascular, respiratory, or systemic disease.

## 2 MATERIALS AND METHODS

### 2.1 Ethics statement

This study was approved by the Texas A&M University and University of Pennsylvania Institutional Animal Care and Use Committees and the Clinical Research Review Committee of the Texas A&M University College of Veterinary Medicine and Biomedical Sciences and included informed owner consent for participation.

### 2.2 Protocol

Seven windows were selected based on described windows that were expected to identify cardiovascular and respiratory abnormalities in the horse. Windows also were selected based on perceived ease of application and execution by non‐experts on horses in various clinical scenarios. Abnormalities sought were pleural effusion, pericardial effusion, moderate or severe lung pathology, pneumothorax, moderate or severe pulmonary hypertension, moderate or severe left heart disease, valvular or myocardial disease, and moderate or severe hypertrophy or pseudohypertrophy of the left ventricle (LV), according to study definitions (Table 1). Standard echocardiographic windows for horses were chosen as recently reviewed,^21^ and lung ultrasound windows were chosen based on literature describing lung sonographic abnormalities in the horse.^22^, ^23^, ^24^ The following are the windows of the protocol and the possible abnormalities to be detected:

- Right parasternal long axis 4‐chamber view (R4C) ‐ The transducer is positioned in the right fourth intercostal space at a level slightly above the olecranon, angled caudally, and rotated clockwise to the 1 o'clock position. The ventricles, atrioventricular valves and atria are imaged. Imaging the LV apex is prioritized and the dorsal aspect of the atrium may not be imaged in its entirety in all horses. This view is used to assess the structures and dimensions of the LV and left atrium (LA). This view also can be used to assess the presence of pericardial effusion, as well as abnormalities of the myocardium and mitral (MV) and tricuspid valves (TV).
- Right parasternal long axis view of the left ventricular outflow tract (LVOT) ‐ Starting from a R4C view, the transducer is angled cranially, and rotated to the 2 o'clock position. It is used to assess the structure and dimensions of the aorta (Ao) and pulmonary artery (PA). Specific attention is paid to the relative size of the PA and the Ao as a marker for pulmonary hypertension. This view also can be used to assess the presence of pericardial effusion, as well as abnormalities of the myocardium and aortic valve (AoV) and TV.
- Right parasternal short axis view at the level of the chordal attachments (SAch). This view is obtained by rotating the transducer clockwise to the 3 to 4 o'clock position from a 4‐chamber view. It is used to assess ventricular size. This view also can be used to assess the presence of pericardial effusion and abnormalities of the myocardium.
- Right parasternal short axis view at the level of the aorta (SAAo) ‐ This view is obtained by moving the transducer dorsally from the SACA and rotating the probe to the 5 to 6 o'clock position. The Aorta (Ao) is seen centrally with the left atrium (LA) and left atrial appendage (LAa) visualized caudally toward the far field. The size of the LA can be directly compared with the Ao, similarly to the LA:Ao ratio described in small animals. This view also can be used to assess the presence of pericardial effusion, as well as abnormalities of the myocardium and AoV.
- Caudoventral thoracic (CAV) window (right and left) ‐ The caudo‐ventral thorax from right side is viewed in a longitudinal plane over the 7th intercostal space. A hyperechoic image of lung, A‐lines and gliding respiratory motions are the expected normal appearance. The diaphragm is viewed at the ventral aspect of the window to ensure ventral position. This view can be used to assess the presence of pleural effusion and lung pathology.
- Caudodorsal thoracic (CAD) window (right and left) ‐ The caudo‐dorsal thorax from right side is viewed in a longitudinal plane over the 15th intercostal space. A hyperechoic image of lung, A‐lines and gliding respiratory motions are the expected normal appearance. This view can be used to assess the presence of pneumothorax, lung pathology and, less commonly, pleural effusion.
- Left parasternal long‐axis 2‐chamber view21 (L2C) ‐ The transducer is positioned in the 5th or 4th left intercostal space slightly above the olecranon. The LV, LA, and MV are viewed. Imaging of the LA is prioritized, and the apex of the LV may not be viewed. This view traditionally has been used for assessment of LA dimensions. This view also can be used to assess the presence of pericardial effusion, as well as abnormalities of the myocardium and MV.

**TABLE 1:** Eight potential abnormalities to be recognized with examination and the criteria for determining the presence or absence.

Abnormality	Definition
1. Is there increased pleural fluid?	Increased pleural fluid is defined as fluid in the pleural space that is more than in the lateral aspect of the most ventral lung tip and more than approximately 1 cm in depth.
2. Is there increased pericardial fluid?	Increased pericardial fluid is defined as fluid visible in the pericardial space that is more than a few millimeters in depth
3. Is there moderate or severe lung pathology such as, consolidation, masses, abscesses and severe or coalescing B‐lines or comet tails.	Consolidation is defined as hypoechoic area of lung with present bronchial or vascular markings. Mass and abscesses are defined as well circumscribed areas in the lung or pleural space that do not contain normal bronchial or vascular markings. Moderate or severe B‐lines or comet tails are defined as interruptions of the smooth and regular hyperechoic echo of lung that merge or create patches.
4. Is there a pneumothorax?	Pneumothorax is the presence of air in the pleural space. This is visible as a hyperechoic echo of gas free in the pleural space (outside of the lung) and therefore the hyperechoic echo of lung is not seen sliding with respiratory motions
5. Is there evidence of moderate or severe pulmonary hypertension?	These are a pulmonary artery that is larger than the aorta's sinotubular junction or shape of the interventricular septum becoming concave right to left
6. Is there evidence of moderate to severe left side heart disease?	These are a left ventricular apex that is rounded and not cone shaped, the left ventricle is severely enlarged and compressing the right ventricle, or the left atrium is moderately or severely enlarged losing its rectangular shape and being disproportionate to the size of the right atrium.
7. Is there moderate or severe left ventricular hypertrophy or pseudohypertrophy?	The left ventricle is thicker in relationship to the left ventricular internal diameter. This corresponds to RWT larger than 0.55.
8. Is there abnormal echogenicity of the myocardium or valves?	This is defined as the presence of hyperechoic, hypoechoic or nodular areas in the myocardium, mitral, tricuspid, or aortic valves.

The proposed CRASH protocol does not define normal ranges for measurement, and sizes are assessed subjectively and relative to internal references. Isopropyl alcohol on unclipped hair was used as a coupling agent. In all images, dorsal or cranial was at the right side of the screen. A pocket‐sized ultrasound unit (Butterfly iQ Vet, Butterfly Network, Guilford, CT) was used with a compatible smart phone device (iPhone 12 Pro, Apple, Cupertino, California). This pocket‐sized device has been used in 2 previous studies in horses that obtained common sonographic windows and detected multiple sonographic abnormalities.^25^, ^26^ Images for this study were acquired in B‐mode with depth set to 30 cm on the “Abdomen Deep” setting, which offers a convex field of view. Images were saved in an online Digital Imaging and Communications in Medicine (DICOM) server. The Butterfly iQ device has only 1 transducer type, and settings are determined by the preset and depth of examination using proprietary algorithms. Imaging windows are described above and in previously publications^23^ and reference images are shown in Figure S1.

### 2.3 Training phase

**FIGURE 1:**
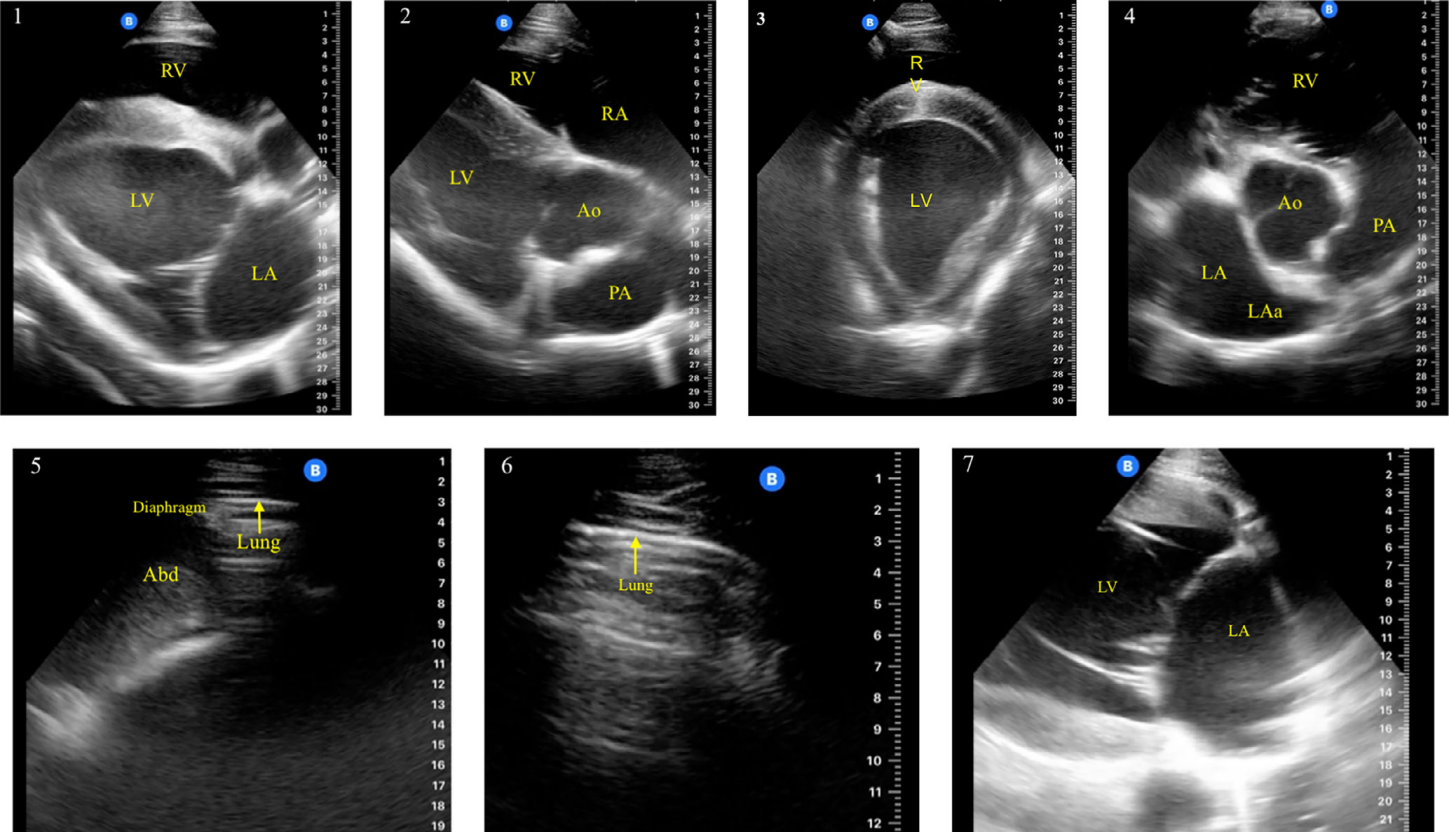
Windows of CRASH. Echocardiographic windows are described as recently summarized by Schwartzwald; thoracic windows are described based on sonographic location of common respiratory diseases in horses.^22^, ^23^, ^24^, ^27^ (1) Right parasternal long axis 4‐chamber view^21^ (R4C, Image 1) ‐ The transducer is positioned in the right fourth intercostal space at a level slightly above the olecranon, angled caudally, and rotated clockwise to the 1 o'clock position. The ventricles, atrioventricular valves and atria are imaged. Imaging the left ventricular (LV) apex is prioritized and the dorsal aspect of the atria may not be imaged in its entirety in all horses. This view is used to assess the structures and dimensions of the LV and left atrium (LA). RA, right atrium; RV, right ventricle. (2) Right parasternal long axis view of the left ventricular outflow tract^21^ (LVOT, Image 2) ‐ Starting from a 4‐chamber view, the transducer is angled cranially, and rotated to the 2 o'clock position. It is used to assess the structure and dimensions of the aorta (Ao) and the pulmonary artery (PA). Specific attention is paid to the relative size of the PA and the Ao as a marker for pulmonary hypertension.^25^, ^28^ LV, left ventricle; RA, right atrium; RV, right ventricle. (3) Right parasternal short axis view at the level of the chordal attachments^21^ (SAch, Image 3). This view is obtained by rotating the transducer clockwise to the 3 to 4 o'clock position from a 4‐chamber view. It is used to assess ventricular size. LV, Left ventricle; RV, right ventricle. (4) Right parasternal short axis view at the level of the aorta^21^ (SAAo, Image 4) ‐ This view is obtained by moving the transducer dorsally from the SAch and rotating the probe to the 5 to 6 o'clock position. The Aorta (Ao) is seen centrally with the left atrium (LA) and left atrial appendage (LAa) visualized caudally toward the far field. The size of the LA can be directly compared with the Ao similarly to the LA:Ao ratio described in small animals.^29^, ^30^, ^31^ PA, pulmonary artery; RV, right ventricle. (5) Caudoventral thoracic^32^ (CAV, Image 5) window (right and left) ‐ The caudo‐ventral thorax from right side is viewed in a longitudinal plane over the 7th intercostal space. A hyperechoic echo of lung, A lines and gliding respiratory motions are the expected normal appearance. The diaphragm is viewed at the ventral aspect of the window to ensure ventral position. (6) Caudodorsal thoracic^32^ (CAD, Image 6) window (right and left) ‐ The caudo‐dorsal thorax from right side is viewed in a longitudinal plane over the 15th intercostal space. A hyperechoic echo of lung, A‐lines and gliding respiratory motions are the expected normal appearance. (7) Left parasternal long‐axis 2‐chamber view^21^ (L2C, Image 7) ‐ The transducer is positioned in the 5th or 4th left intercostal space slightly above the olecranon. The left ventricle (LV), left atrium (LA), and mitral valve are viewed. Imaging of the LA is prioritized, and the apex of the LV may not be viewed. This view has traditionally been used for assessment of LA dimensions.

During the study period, sonographer 1 recently had completed a large animal training program approved by the American College of Veterinary Internal Medicine (ACVIM) and was performing a 1‐year equine cardiology fellowship sponsored by the ACVIM. Sonographer 2 was an expert sonographer who was board‐certified by the ACVIM in large animal internal medicine and had 10 years of experience in ultrasonography of horses and cardiology including fellowship training in equine cardiology. The training phase was performed to determine if the pocket‐sized device could acquire good quality images for the CRASH protocol. The training phase also allowed sonographer 1 to become familiar with the device and the protocol and to receive input from sonographer 2 on skills for acquiring images. Input included optimizing image quality and optimizing visualization of anatomic structures of interest. This input was offered after the protocol was completed and when images were reviewed. Ten horses were enrolled: 9 Thoroughbreds and 1 Standardbred. For each horse, sonographer 1 and 2 separately obtained and saved video clips of the windows of the CRASH protocol with pocket‐sized equipment. The protocol then was performed by sonographer 2 using a standard echocardiographic ultrasound system (Vivid E95 Cardiac Ultrasound, M5Sc‐D sector transducer, 1.5 to 4.5 MHz, General Electric, USA). Sonographer 2 then evaluated images obtained by sonographer 1 and determined them to be of either acceptable or unacceptable quality. An image was considered unacceptable if anatomic structures described in Figure 1 were not visible (see Table 1) and would not have enabled answering the clinical questions described in Table 1. An image was considered acceptable if anatomic structures were visible and the image would enable a sonographer to answer the questions described in Table 1. Sonographers 1 and 2 reviewed images obtained using the pocket‐sized device and standard ultrasound machine to gain insights about variations associated with individual horse factors, device capability, or operator error, and to subsequently improve the image quality.

### 2.4 Feasibility phase

After completing the training phase, a prospective, descriptive study was undertaken to determine if the CRASH protocol could be performed in 3 different clinical scenarios and what duration of time would be required to complete the protocol. Sonographer 1 performed the CRASH protocol in various settings which included: healthy horses free of signs of cardiac, respiratory, or systemic disease (healthy group; n = 27), horses performing in athletic events (athletic group; n = 14), and client‐owned horses admitted to the teaching hospital with signs of cardiac, respiratory, or systemic disease (hospital group; n = 50). Horses aged ≥1 year were eligible for inclusion; miniature horses and donkeys were excluded. Horses with systemic disease were included because of the prevalence of cardiorespiratory disease in these horses and to assess the ability of CRASH to detect echocardiographic abnormalities reported to be associated with systemic disease such as volume depletion (pseudohypertrophy of the LV) or hypertension (hypertrophy of the LV).^33^, ^34^ Horses in the healthy group were determined to be healthy based on history and physical examination and were part of a university‐owned teaching herd and university equestrian team. Horses were standing and restrained with a halter and lead rope by an experienced handler or placed in stocks. For the athletic group, horses participating in equestrian events (athletic group) were enrolled. Horses were assessed at the event facility either before or immediately after the competitive event. Horses were standing and restrained with a halter and lead rope either by the owner or while tied. Tack was left in place based on the owner's preference. Horses in the hospital group were those that presented to the 2 collaborating institutions during the study period when both sonographers were available for clinical duties. Horses presented on an emergency or elective basis with any of the following were enrolled in the hospital group with owner consent: arrhythmia (diagnosed electrocardiographically), unexplained tachycardia (heart rate > 52 beats per minute), cardiac murmur' increased respiratory effort or dyspnea, coughing, fever (temperature > 38.6°C [>101.5°F]), or signs of abdominal pain (colic). Horses were standing and restrained with a halter and lead rope or placed in stocks for examination. Priority was given to assessment of the presenting complaint by the primary clinician, and therefore patient location was dependent on orders from the clinician on the case and not the primary investigator. The duration of the sonogram was the time between the first and last obtained images. For this phase, the review process involved identifying normal anatomical structures that typically are observed within each window (Figure 1). Sonographer 2 then evaluated clips obtained by sonographer 1 and determined them to be of either acceptable or unacceptable quality. An image was considered unacceptable if anatomic structures described in Figure 1 were not visible and would not have enabled answering the clinical questions described in Table 1. An image was considered acceptable if anatomic structures were visible and the image would enable a sonographer to answer the questions described in Table 1. Sonographer 2 was not blinded to how the horse was grouped (healthy, hospitalized, or athletic). For horses that underwent >1 CRASH sonogram during hospitalization, only the first examination was included.

### 2.5 Clinical phase

To identify the abnormalities detected with the CRASH protocol, stored video clips from the hospital group (n = 50) were evaluated by sonographer 2, who was considered an expert in ultrasonography of horses based on previously mentioned credentials, for the presence or absence of 8 predefined cardiovascular or respiratory abnormalities (Table 1). The CRASH examinations performed by sonographer 2 on 70 additional hospitalized horses that met the same inclusion criteria as the feasibility study hospital group were included. Stored video clips from the 70 additional cases were evaluated for presence or absence abnormalities described in Table 1 after completion of the study by sonographer 2. The objective of the clinical phase was to evaluate whether predefined abnormalities can be detected by an expert sonographer using the CRASH protocol. Sonographer 2 was not blinded to the clinical signs for which the hospitalized horses were presented.

### 2.6 Data analysis

Descriptive statistics were used to summarize data. Continuous data were summarized as means and SDs, and 95% confidence intervals (95% CI) were estimated using exact methods; ordinal data were summarized as medians and ranges. Categorical data were summarized using proportions. Descriptive statistics were calculated using R statistical software (Version 3.3.3, R Core Team, Vienna, Austria).

## 3 RESULTS

### 3.1 Training phase

For all horses of a homogenous group, sonographers 1 and 2 were able to obtain images in all windows in all 10 horses, and all images obtained in each window were considered of acceptable quality. The operator using a POCUS device was able to obtain acceptable images consistently.

### 3.2 Feasibility study

Twenty‐seven healthy horses were enrolled in the healthy horse group. Various breeds were represented (20 Quarter Horses, 6 Thoroughbreds, 1 Warmblood) with a mean age of 5 years (SD, 5 years) and body condition scores (BCS) ranged from 4 to 7 out of 9 with a median of 5 (Figure S2).^35^ Fourteen horses were enrolled in the athletic group, of which 7 were Quarter Horses performing at a rodeo event and 7 were horses performing at a polo scrimmage. The mean age of the horses in the athletic group was 18 years (SD, 4 years). Body condition score ranged from 4 to 5 out of 9 with a median of 5. Fifty horses were enrolled in the hospital group for the feasibility portion of the study, including 20 Quarter Horses (44%), 11 Thoroughbreds (22%), 5 draft horse breeds (10%), 4 Warmbloods (8%), 4 mixed‐breed horses (8%), 3 Paint Horses (6%), and 1 each of Morgan, Andalusian, and Arabian. The mean age of the hospital group was 14 years (SD, 8 years). Body condition score ranged from 2 to 7 out of 9, with a median of 5. The clinical findings that prompted focused cardiorespiratory ultrasound examination included abdominal pain (n = 15), murmur (n = 12; systolic, 7, diastolic, 5), coughing or tachypnea (n = 10), arrhythmia (n = 8; atrial fibrillation, 7, ventricular premature complex, 1), fever (n = 3), and unexplained tachycardia (n = 2). Twenty‐five of the horses were presented on an elective basis where the other 25 were presented on an emergency basis. Five of the 50 hospital horses met criteria for enrollment based on incidental findings (murmur) whereas the other 45 horses were presented for a primary problem that resulted in at least 1 of the criteria for enrollment. The percentage of images of acceptable quality obtained per window for each group and the 95% CI and mean (± SD) of the duration of sonograms for these horses were tabulated (Table 2). All thoracic windows provided acceptable quality images in athletic horses and normal horses, which were a homogenous group of horses maintained in a similar environment. The 8% of hospitalized horses that had poor quality thoracic windows were horses in which none of the images of the CRASH protocol were acceptable. The caudodorsal thoracic windows could not be obtained in the 7 horses participating in rodeo events because of western tack being placed over the sonographic window, and therefore these windows were not included in the analysis. Regarding the cardiac windows, for healthy and hospitalized horses the 4‐chamber long axis window was most consistently of good quality (92.5%, 82% respectively). The short axis at the level of the aorta had the lowest percentage of acceptable quality images in the normal (74.1%) and hospitalized (60%) groups. For the athletic horses, the short axis at the level of the chordal attachments had the lowest percentage of acceptable quality images (78.7%) and the 2‐chamber long axis window was most consistently of acceptable quality (100%); all other cardiac windows also had a high percentage of acceptable quality images.

**TABLE 2:** Percentage of acceptable quality images based on review by sonographer 2 for each of the windows obtained by sonographer 1; duration of time required for examination by sonographer 1.

Window	Healthy group (n = 27)	Hospital group (n = 50)	Athletic group (n = 14)
R4C	92% (CI: 76%‐99%)	82% (CI: 69%‐91%)	93% (CI: 66%‐100%)
LVOT	85% (CI: 66%‐96%)	82% (CI: 69%‐91%)	93% (CI: 66.1%‐99.8%)
SAch	89% (CI: 71%‐97.6%)	68% (CI: 54%‐80%)	79% (CI: 49%‐95%)
SAAo	74% (CI: 54%‐89%)	60% (CI: 45%‐74%)	93% (CI: 66%‐100%)
L2C	74.% (CI: 54%‐89%)	76% (CI: 62%‐86.9%)	100% (CI: %‐100.0%)
CAV	100% (CI: 87%‐100%)	92% (CI: 80%‐98%)	100%^a^ (CI: 77%‐100%)
CAD	100% (CI: 87%‐100%)	92% (CI: 80%‐98%)	100% (CI: 59%‐100%)
Duration	5 min 42 s ± 1 min 29 s	6 min 54 s ± 1 min 59 s	5 min 30 s ± 56 s

Abbreviations: CAD, Caudodorsal thoracic view; CAV, Caudoventral thoracic view; CI, 95% confidence interval; L2C, 2‐chamber long axis view; LVOT, left ventricular outflow tract view; R4C, four chamber long‐axis view; SAAo, short axis view at the level of the aorta; SAch, short‐axis view at the chordal attachments.

^a^ Only 7 of the athletic horses had CAD images because of the placement of tack.

### 3.3 Clinical phase

Sonographic abnormalities identified in horses (n = 120) presented with cardiac, respiratory, or systemic disease were tabulated (Table 3). These horses included 50 horses examined for the feasibility study by sonographer 1 at 2 different institutions (34 horses examined at Texas A&M Veterinary Medical Teaching Hospital and 16 horses examined at University of Pennsylvania's New Bolton Center) and 70 additional horses examined by sonographer 2 at University of Pennsylvania's New Bolton Center. The 70 additional horses included 45 geldings, 19 mares, and 6 stallions. There were 22 Warmbloods (31%), 17 Thoroughbreds (24%), 9 Standardbreds (13%), 7 mixed‐breed horses (10%), 5 Quarter Horses (7%), 2 Morgans (3%), 2 Arabians (3%), 2 draft horse breeds (3%), and 1 each (1%) of Paint Horse, Appaloosa, Welsh Phony, and Rocky Mountain Horse breeds. The mean age of the hospital group was 10 years (SD ± 5 years). Body condition score was not recorded. The clinical findings that prompted focused cardiorespiratory ultrasound examination included murmur (n = 25), fever (n = 19), arrhythmia (n = 17), coughing or tachypnea (n = 13), abdominal pain (n = 12), and unexplained tachycardia (n = 3). Horses with >1 qualifying abnormality were enrolled. For horses with >1 abnormality detected with CRASH or for which an abnormality was detected in >1 window, all detected abnormalities and windows were described (Table 3; Figure S3). For the 70 additional horses examined by sonographer 2, the duration of the sonogram was a mean of 5.9 min (SD ± 5.8 min) and the duration of the sonogram for horses imaged by sonographer 1 in the feasibility study was a mean of 6.9 min (SD ± 2.0 min). In 3 of the 70 horses (4%), no cardiac images of diagnostic quality could be obtained.

**TABLE 3:** Sonographic abnormalities identified in horses (n = 120) presented with cardiac, respiratory, or systemic disease.

Abnormal finding	Number of cases with abnormality	Window where abnormality observed
1. Pleural fluid	15	1‐R4C, 1‐SAch, 15‐CAV, 1‐CAD
2. Pericardial fluid	1	R4C, LVOT, SAch, SAAo
3. Lung pathology	a ‐ coalescing comet tails: 14 b ‐ consolidation: 10	9‐CAV, 4‐CAD, 1‐R4C, 1‐L2C 10‐CAV
4. Pneumothorax	2	2‐CAD
5. Pulmonary hypertension	7	3‐R4C, 7‐LVOT, 3‐SAch, 1‐SAAo
6. Left heart disease	14	14‐R4C, 6‐LVOT, 11‐SAch, 3‐SAAo, 5‐L2C
7. Valvular or myocardial disease	a ‐ valvular: 1 b ‐ myocardial: 2	1‐R4C, 3‐LVOT, 1‐SAch, 2‐SAAo
8. Hypertrophy or pseudohypertrophy	8	8‐R4C, 8‐SAch, 2‐L2C
No significant findings	62	NA

*Note*: Findings were identified based on definitions in Table 1 by sonographer 2. Windows are labeled as described in Figure 1.

Abbreviations: CAD, Caudodorsal thoracic view; CAV, Caudoventral thoracic view; L2C, two chamber long axis view; LVOT, left ventricular outflow tract view; R4C, four chamber long‐axis view; SAAo, short axis view at the level of the aorta; SAch, short‐axis view at the chordal attachments.

## 4 DISCUSSION

Our results indicated that performing the proposed CRASH protocol was feasible using a pocket‐sized ultrasound device in healthy, hospitalized, and athletic horses. The time required to complete the examination for all groups was suitable for most time‐sensitive scenarios.

Thoracic windows were most consistently acquired and of clinically acceptable quality. Horses that had poor quality thoracic windows were those in which none of the windows of CRASH were of good quality. Increased body condition, long hair coat, or both often were recorded, but the association of these patient findings with the quality of images was not statistically analyzed (because it was not an a priori hypothesis). Our perception is that these patient factors contributed to poor image quality. Horses were not clipped for examination, which was an intentional decision to respect a time‐sensitive clinical scenario in which focused examinations often are used. Clipping of the hair might have improved image quality for some of these cases. It is our opinion that thoracic images are less technically challenging as compared to echocardiographic windows, which also may have contributed to the increase in percentage of good quality thoracic images versus cardiac images. Echocardiographic right parasternal long axis views were obtained on average in >80% of cases whereas short axis views and left parasternal echocardiographic views of clinically acceptable quality were obtained less frequently. All clinical abnormalities identified in the 120 stored CRASH examinations in horses with clinical disease would have been detected by a combination of R4C, LVOT, CAV, and CAD windows. The American Society of Echocardiography recommends that, in ideal situations and when patient condition allows, anatomic structures of interest should be visualized in at least 2 different sonographic windows when using POCUS protocols. The need for this approach in common clinical diseases of horses and clinical scenarios will need to be tested prospectively. It is probable that a simplified protocol limited to thoracic and long axis right parasternal views would be easier to teach and simpler to interpret and therefore offer the best combination of (a) ease of training novice sonographers to complete and interpret CRASH and (b) ability to provide clinically useful information. It is our impression that short axis views, and more commonly the SAch, help increase the confidence in findings identified in a R4C view, but this perception would need to be further evaluated.

Focused cardiac and thoracic ultrasound examinations are increasingly performed across a range of human and small animal medical specialties and can be performed with good diagnostic performance by clinicians (CPUS) that are not cardiologists, internal medicine specialists or radiologists.^11^ A limitation of our study was that only 1 sonographer performed the CRASH protocol during the feasibility phase. Therefore, this phase was also an evaluation of the sonographer's skill and not just an evaluation of the protocol. The sonographer‐in‐training (sonographer 1) had completed a large animal internal medicine residency, became board‐certified by the American College of Veterinary Internal Medicine (LAIM) during the time span of the project, and was in a large animal cardiology clinical training fellowship program approved and supported by the ACVIM. Ultrasound skills of individuals undergoing training have been reported to improve until approximately 300 examinations are completed at which time skills start to plateau.^36^ At the time of the study, sonographer 1 had not achieved this number and it is reasonable to believe that skills were developing but above average for the general population of equine practitioners who might use CRASH. In a recent study, participants who were non‐specialist practitioners learned to perform and interpret a focused echocardiographic protocol for horses independent of previous experience in a 1‐day course using Peyton's 4‐step approach.^20^

The described protocol was feasible in horses participating in equestrian events. Similarly, the use of focused cardiac ultrasound by sports medicine physicians has proved successful for acquiring images that recognize common causes of sudden athletic death such as hypertrophic cardiomyopathy and aortic root dilatation in human athletes.^37^, ^38^ The incidence of sudden athletic death in horses that participate in high intensity exercise disciplines is estimated to be over 200 times higher than that of humans.^39^, ^40^ Echocardiography is considered a second‐tier diagnostic modality in pre‐participation screening programs for human athletes, where history, physical examination, and 12‐lead ECG are the most common components of the programs.^41^ The conditions that cause sudden athletic death in horses and humans are different^42^ and therefore it is plausible that prevention likely requires a different approach. The ability of a protocol such as that described here to detect preventable causes of sudden athletic death requires further study.

The hospitalized group of horses had the lowest percentage of good quality images for all windows except L2C. It is our impression that some factors associated with hospitalized horses may have influenced the ability to acquire good quality images such as patient positioning, severity of pain, or concurrent performance of other procedures (eg, passage of nasogastric tube, placement of IV catheter). This group of horses was included in the study with recognition that these factors are likely to be present in common clinical scenarios in which the CRASH may be used. Cardiac short axis (SAAo and SAch) and left parasternal views (L2C) frequently had a lower percentage of acceptable quality images (Table 2). It is possible these images are technically more demanding to acquire in the horse.

The targeted CRASH protocol described here was designed with critical care and triage scenarios in mind. It aims to provide information to aid decisions about the need for additional diagnostic testing, timely referral for specialist evaluation, or therapeutic intervention. Cases with moderate or severe disease were the target of this protocol. Thus, this CRASH protocol may not be suitable for the examination of all cardiorespiratory cases, nor is it intended to replace examination by specialists using a comprehensive thoracic ultrasound examination or echocardiogram when indicated. Specific windows were chosen based on their perceived high likelihood to yield specific abnormalities. Abnormalities identified included pleural effusion, pericardial effusion, lung consolidation, coalescing B‐lines or comet tail artifacts, pneumothorax, pulmonary hypertension, myocardial and valvular abnormal echogenicity, moderate‐to‐severe left‐sided heart disease, or cardiac hypertrophy or pseudohypertrophy. The abnormalities detected were likely influenced by the study population as well as the higher incidence of respiratory disease as compared to cardiac disease in horses. Abnormalities are not always detected in the acoustic windows described for CRASH and it is therefore possible that certain abnormalities might go unrecognized using the described protocol. Clinicians performing CRASH should be aware of these limitations and not consider the examination a replacement for more comprehensive diagnostic testing or specialist evaluation. Also, the abnormalities detected with CRASH were not formally compared to findings of more comprehensive tests. A different study comparing results of the CRASH protocol to gold standards will be needed to accomplish this necessary step.

A limitation of the study was that a sonographer who was considered an expert evaluated images to determine the presence or absence of certain abnormalities. This sonographer also performed a portion of the CRASH examinations during the clinical phase. This approach may have led to the detection of abnormalities that might not have been detectable by a practitioner with less experience because ultrasound examination is a highly operator‐dependent modality both for acquisition and interpretation of images. Evaluation of training programs is warranted to ensure operators using POCUS have the necessary skill and knowledge to use the examination effectively. Also, the influence of experience in the diagnostic accuracy of the CRASH protocol described here in different groups of operators should be systematically evaluated in future studies.

## 5 CONCLUSIONS

We described a focused cardiorespiratory ultrasound protocol (CRASH) that could be performed in healthy and diseased horses in a hospital and barn setting in 5.5 to 6.9 min on average. A sonographer in training could acquire good quality images most consistently through thoracic windows followed by right parasternal long axis echocardiographic windows. Abnormalities that were commonly detected with the CRASH protocol by an expert sonographer included pleural fluid, lung consolidation, coalescing B‐lines or comet tail artifacts, and moderate or severe left heart disease. Sonographic evidence of moderate or severe pulmonary hypertension or left ventricular hypertrophy or pseudohypertrophy also were common findings in our study population, whereas pericardial fluid, pneumothorax or moderate or severe myocardial or valvular echogenicity changes were detected rarely. The diagnostic accuracy and potential clinical utility of the CRASH protocol to improve veterinary care of horses deserves further evaluation.

## CONFLICT OF INTEREST DECLARATION

Authors declare no conflict of interest.

## OFF‐LABEL ANTIMICROBIAL DECLARATION

Authors declare no off‐label use of antimicrobials.

## INSTITUTIONAL ANIMAL CARE AND USE COMMITTEE (IACUC) OR OTHER APPROVAL DECLARATION

Approved by the Texas A&M University IACUC and the Clinical Research Review Committee of the Texas A&M College of Veterinary Medicine & Biomedical Sciences (Protocol #2020‐0069 CA) and included informed owner consent for participation. Also approved by the University of Pennsylvania IACUC (Protocol #‐ POAP‐806975).

## HUMAN ETHICS APPROVAL DECLARATION

Authors declare human ethics approval was not needed for this study.

## Supporting information


**FIGURE S1.** CRASH feasibility study

**FIGURE S2.** Body condition scores (range, 1‐9) for horses in feasibility phase of study.

**FIGURE S3.** Images from horses presented for systemic disease and abnormalities detected in CRASH examination. 1. Right parasternal long axis 4‐chamber view (R4C) ‐ There is severe left atrial enlargement. The left atrium is rounded, several fold larger than the right atrium and expanded beyond the far field of a 30 cm displayed depth. Left Ventricle = LV, Left atrium = LA, Right ventricle = RV, Right atrium = RA. 2. Right parasternal long axis 4‐chamber view (R4C) ‐ The myocardium of the left ventricle is subjectively thickened (star) with an appearance of hypertrophy/pseudohypertrophy. Left Ventricle = LV, Left atrium = LA, Right ventricle = RV, Right atrium = RA. 3. Right parasternal long axis view of the left ventricular outflow tract (LVOT) ‐ The pulmonary artery is larger than the aorta and the interventricular septum is flattened convex right to left suggesting pulmonary hypertension. Aorta = Ao, Pulmonary artery = PA. Left Ventricle = LV, Right ventricle = RV, Interventricular Septum = IVS. 4. Right parasternal long axis view of the left ventricular outflow tract (LVOT) ‐ The aorta has a large nodular lesion (arrow). Aorta = Ao, Pulmonary artery = PA. Left Ventricle = LV, Right ventricle = RV. 5. Right parasternal short axis view at the level of the chordal attachments (SAch) ‐ The myocardium of the left ventricle is subjectively thickened (double arrow) with an appearance of hypertrophy/pseudohypertrophy. Left Ventricle = LV, Right ventricle = RV. 6. Right parasternal short axis view at the level of the aorta (SAAo) ‐ The size of the LA is disproportionally large in comparison with the size of the Ao. Aorta = Ao, Left Atrium = LA, Right ventricle = RV, Pulmonary artery = PA 7. Caudodorsal (CAD) thoracic window (right and left) ‐ A hyperchoic echo of lung with coalescing B lines/comet tail artifacts is seen. 8. Caudoventral thoracic (CAV) window (right and left) ‐ There is a large amount of pleural fluid. Pleural effusion = PE, Diaphragm = Di.

**FIGURE S4.** Abnormalities detected with CRASH and the clinical finding(s) that prompted CRASH evaluation for Clinical Phase. Number of cases that had abnormality listed in left column (n=) along with abnormality. Some cases had more than 1 clinical finding that met criteria for enrollment in the clinical phase.

## References

[1] Baston CM , Wallace P , Chan W , Dean AJ , Panebianco N . Innovation through collaboration: creation of a combined emergency and internal medicine point‐of‐care ultrasound fellowship. J Ultrasound Med. 2019;38:2209‐2215. 30592332 10.1002/jum.14908

[2] Luong CL , Ong K , Kaila K , Pellikka PA , Gin K , Tsang TSM . Focused cardiac ultrasonography current applications and future directions. J Ultrasound Med. 2018;38:865‐876. 30146784 10.1002/jum.14773

[3] Volpicelli G , Elbarbary M , Blaivas M , et al. For the International Liaison Committee on Lung Ultrasound (ILC‐LUS) for International Consensus Conference on Lung Ultrasound (ICC‐LUS). International evidence‐based recommendations for point‐of‐care lung ultrasound. Intensive Care Med. 2012;38:577‐591. 22392031 10.1007/s00134-012-2513-4

[4] Xue H , Li C , Cui L , et al. M‐BLUE protocol for coronavirus disease‐19 (COVID‐19) patients: interobserver variability and correlation with disease severity. Clin Radiol. 2021;76:379‐383. 33663912 10.1016/j.crad.2021.02.003 PMC7888246

[5] Liu J , Copetti R , Sorantin E , et al. Protocol and guidelines for point‐of‐care lung ultrasound in diagnosing neonatal pulmonary diseases based on international expert consensus. J Vis Exp. 2019;6:1‐20. 10.3791/58990 30907892

[6] Bergmann KR , Arroyo AC , Tessaro MO , et al. Diagnostic accuracy of point‐of‐care ultrasound for intussusception: a multicenter, noninferiority study of paired diagnostic tests. Ann Emerg Med. 2021;21:606‐615. 10.1016/j.annemergmed.2021.04.033 34226072

[7] Hori Y , Yamashita Y , Sakakibara K , Sano T , Hori A . Usefulness of pericardial lung ultrasonography for the diagnosis of cardiogenic pulmonary edema in dogs. Am J Vet Res. 2020;81:227‐232. 32101047 10.2460/ajvr.81.3.227

[8] Vientos‐Plotts AI , Wiggen KE , Lisciandro GR , et al. The utility of point‐of‐care ultrasound right‐sided cardiac markers as a screening test for moderate to severe pulmonary hypertension in dogs. Vet J. 2019;250:6‐13. 31383421 10.1016/j.tvjl.2019.05.013

[9] Darnis E , Merveille AC , Desquilbet L , Boysen S , Gommeren K . Interobserver agreement between non‐cardiologist veterinarians and a cardiologist after a 6‐hour training course for echographic evaluation of basic echocardiographic parameters and caudal vena cava diameter in 15 healthy Beagles. J Vet Emerg Crit Care. 2019;29:495‐504. 10.1111/vec.12883 31453666

[10] Oricco S , Rabozzi R , Meneghini C , Franci P . Usefulness of focused cardiac ultrasonography for predicting fluid responsiveness in conscious, spontaneously breathing dogs. Am J Vet Res. 2019;80:369‐377. 30919671 10.2460/ajvr.80.4.369

[11] Loughran KA , Rush JE , Rozanski EA , Oyama MA , Larouche‐Lebel É , Kraus MS . The use of focused cardiac ultrasound to screen for occult heart disease in asymptomatic cats. J Vet Intern Med. 2019;33:1892‐1901. 31317580 10.1111/jvim.15549 PMC6766524

[12] Tse YC , Rush JE , Cunningham SM , Bulmer BJ , Freeman LM , Rozanski EA . Evaluation of a training course in focused echocardiography for noncardiology house officers. J Vet Emerg Crit Care. 2013;23:268‐273. 10.1111/vec.12056 23647602

[13] Diamantino AC , Nascimento BR , Nunes MCP , et al. Impact of incorporating echocardiographic screening into a clinical prediction model to optimise utilisation of echocardiography in primary care. Int J Clin Pract. 2021;75:1‐5. 10.1111/ijcp.13686 32852108

[14] Mantuan D , Frazee BW , Fahimi J , et al. Point‐of‐care multi‐organ ultrasound improves diagnostic accuracy in adults presenting to the emergency department with acute dyspnea. West J Emerg Med. 2016;17:46‐53. 26823930 10.5811/westjem.2015.11.28525 PMC4729418

[15] Spencer KT , Kimura BJ , Korcarz CE , Pellikka PA , Rahko PS , Siegel RJ . Focused cardiac ultrasound: recommendations from the American society of echocardiography. J Am Soc Echocardiogr. 2013;26:567‐581. 23711341 10.1016/j.echo.2013.04.001

[16] Labovitz AJ , Noble VE , Bierig M , et al. Focused cardiac ultrasound in the emergent setting: a consensus statement of the American Society of Echocardiography and American College of Emergency Physicians. J Am Soc Echocardiogr. 2010;23:1225‐1230. 21111923 10.1016/j.echo.2010.10.005

[17] Via G , Hussain A , Wells M , et al. International evidence‐based recommendations for focused cardiac ultrasound. J Am Soc Echocardiogr. 2014;27:683.e1‐e33. 10.1016/j.echo.2014.05.001 24951446

[18] Busoni V , De Busscher V , Lopez D , et al. Evaluation of a protocol for fast localised abdominal sonography of horses (FLASH) admitted for colic. Vet J. 2011;188:77‐82. 20347357 10.1016/j.tvjl.2010.02.017

[19] Vincze B , Baska F , Papp M , Szenci O . Introduction of a new fetal examination protocol for on‐field and clinical equine practice. Theriogenology. 2019;125:210‐215. 30469011 10.1016/j.theriogenology.2018.11.004

[20] Eberhardt C , Schwarzwald CC . Focused cardiac ultrasound examination in the emergency and critical care equine patient: training for non‐specialist veterinarians and evaluation of proficiency. J Vet Intern Med. 2022;36:1471‐1480. 35657023 10.1111/jvim.16446 PMC9308440

[21] Schwarzwald CC . Equine echocardiography. Vet Clin North Am Equine Pract. 2019;35:43‐64. 30826106 10.1016/j.cveq.2018.12.008

[22] Partlow J , David F , Hunt LM , et al. Comparison of thoracic ultrasonography and radiography for the detection of induced small volume pneumothorax in the horse. Vet Radiol Ultrasound. 2017;58:354‐360. 28264227 10.1111/vru.12480

[23] Byars TD , Becht JL . Pleuropneumonia. Vet Clin North Am Equine Pract. 1991;7:63‐78. 2054710 10.1016/s0749-0739(17)30516-3

[24] Reimer JM , Reef VB , Spencer PA . Ultrasonography as a diagnostic aid in horses with anaerobic bacterial pleuropneumonia and/or pulmonary abscessation: 27 cases (1984‐1986). J Am Vet Med Assoc. 1989;194:278‐282. 2645254

[25] Deacon LJ , Reef VB , Leduc L , Navas de Solis C . Pocket‐sized ultrasound versus traditional ultrasound images in equine imaging: a pictoral essay. J Equine Vet Sci. 2021;104:103672. 34416998 10.1016/j.jevs.2021.103672

[26] Williams ZJ , Sage A , Valberg SJ . Hand‐held point‐of‐care ultrasound: a new tool for veterinary student self‐driven learning in the time of COVID‐19. J Vet Med Edu. 2022;3:306‐311. 10.3138/jvme-2020-0131 33970815

[27] Decloedt A , Borowicz H , Slowikowska M , Chiers K , Loon G , Niedzwiedz A . Right ventricular function during acute exacerbation of severe equine asthma. Equine Vet J. 2017;49:603‐608. 28132404 10.1111/evj.12675

[28] Johansson AM , Gardner SY , Atkins CE , LaFevers DH , Breuhaus BA . Cardiovascular effects of acute pulmonary obstruction in horses with recurrent airway obstruction. J Vet Intern Med. 2007;21:302‐307. 17427392 10.1892/0891-6640(2007)21[302:ceoapo]2.0.co;2

[29] Keene BW , Atkins CE , Bonagura JD , et al. ACVIM consensus guidelines for the diagnosis and treatment of myxomatous mitral valve disease in dogs. J Vet Intern Med. 2019;33:1127‐1140. 30974015 10.1111/jvim.15488 PMC6524084

[30] Huesler I , Mitchell K , Schwarzwald C . Echocardiographic assessment of left atrial size and function in Warmblood horses: reference intervals, allometric scaling, and agreement of different echocardiographic variables. J Vet Intern Med. 2016;30:1241‐1252. 27362277 10.1111/jvim.14368 PMC5108425

[31] Marr CM . Equine acquired valvular disease. Vet Clin North Am Equine Pract. 2019;35:119‐137. 30871831 10.1016/j.cveq.2018.12.001

[32] Reef VB . Thoracic ultrasonography: noncardiac imaging. In: Reef VB , ed. Equine Diagnostic Ultrasound. Vol 1. Philadelphia, PA: Elsevier Saunders; 1998:187‐214.

[33] Underwood C , Norton J , Nolen‐Walston R , et al. Echocardiographic changes in heart size in hypohydrated horses. J Vet Intern Med. 2011;25:563‐569. 21039870 10.1111/j.1939-1676.2010.0612.x

[34] Navas de Solis C , Slack J , Boston RC , Reef VB . Hypertensive cardiomyopathy in horses: 5 cases (1995‐2011). J Am Vet Med Assoc. 2013;243(1):126‐130. 23786201 10.2460/javma.243.1.126

[35] Henneke D , Potter G , Kreider J , Yeates B . Relationship between condition score, physical measurements and body‐fat percentage in mares. Equine Vet J. 1983;15:371‐372. 6641685 10.1111/j.2042-3306.1983.tb01826.x

[36] Duanmu Y , Henwood PC , Takhar SS , et al. Correlation of OSCE performance and point‐of‐care ultrasound scan numbers among a cohort of emergency medicine residents. Ultrasound J. 2019;11:1‐5. 31359167 10.1186/s13089-019-0118-7 PMC6638613

[37] Yim ES , Kao D , Gillis EF , Basilico FC , Corrado GD . Focused physician‐performed echocardiography in sports medicine: a potential screening tool for detecting aortic root dilatation in athletes. J Ultrasound Med. 2013;32:2101‐2106. 24277891 10.7863/ultra.32.12.2101

[38] Yim ES , Gillis EF , Ojala K , MacDonald J , Basilico FC , Corrado GD . Focused transthoracic echocardiography by sports medicine physicians: measurements relevant to hypertrophic cardiomyopathy. J Ultrasound Med. 2013;32:333‐338. 23341391 10.7863/jum.2013.32.2.333

[39] Maron BJ , Doerer JJ , Haas TS , Tierney DM , Mueller FO . Sudden deaths in young competitive athletes: analysis of 1866 deaths in the United States, 1980‐2006. Circulation. 2009;119:1085‐1092. 19221222 10.1161/CIRCULATIONAHA.108.804617

[40] Physick‐Sheard PW , Slack J . Irregular hearts and performance horses. Equine Vet J. 2020;52:782‐786. 33017494 10.1111/evj.13316

[41] Corrado D , Pelliccia A , Bjørnstad HH , et al. Consensus Statement of the Study Group of Sport Cardiology of the Working Group of Cardiac Rehabilitation and Exercise Physiology and the Working Group of Myocardial and Pericardial Diseases of the European Society of Cardiology. Eur Heart J. 2005;26:516‐524. 15689345 10.1093/eurheartj/ehi108

[42] de Solis CN , Althaus F , Basieux N , et al. Sudden death in sport and riding horses during and immediately after exercise: a case series. Equine Vet J. 2018;50:644‐648. 29330860 10.1111/evj.12803

