# Supplementary figures and images for: Feasibility of a point‐of‐care ultrasound protocol for cardiorespiratory evaluation of horses in different clinical settings
Source: J Vet Intern Med. 2023 Mar 28;37(3):1223–32. doi: 10.1111/jvim.16674 (PMC10229348; doi:10.1111/jvim.16674)

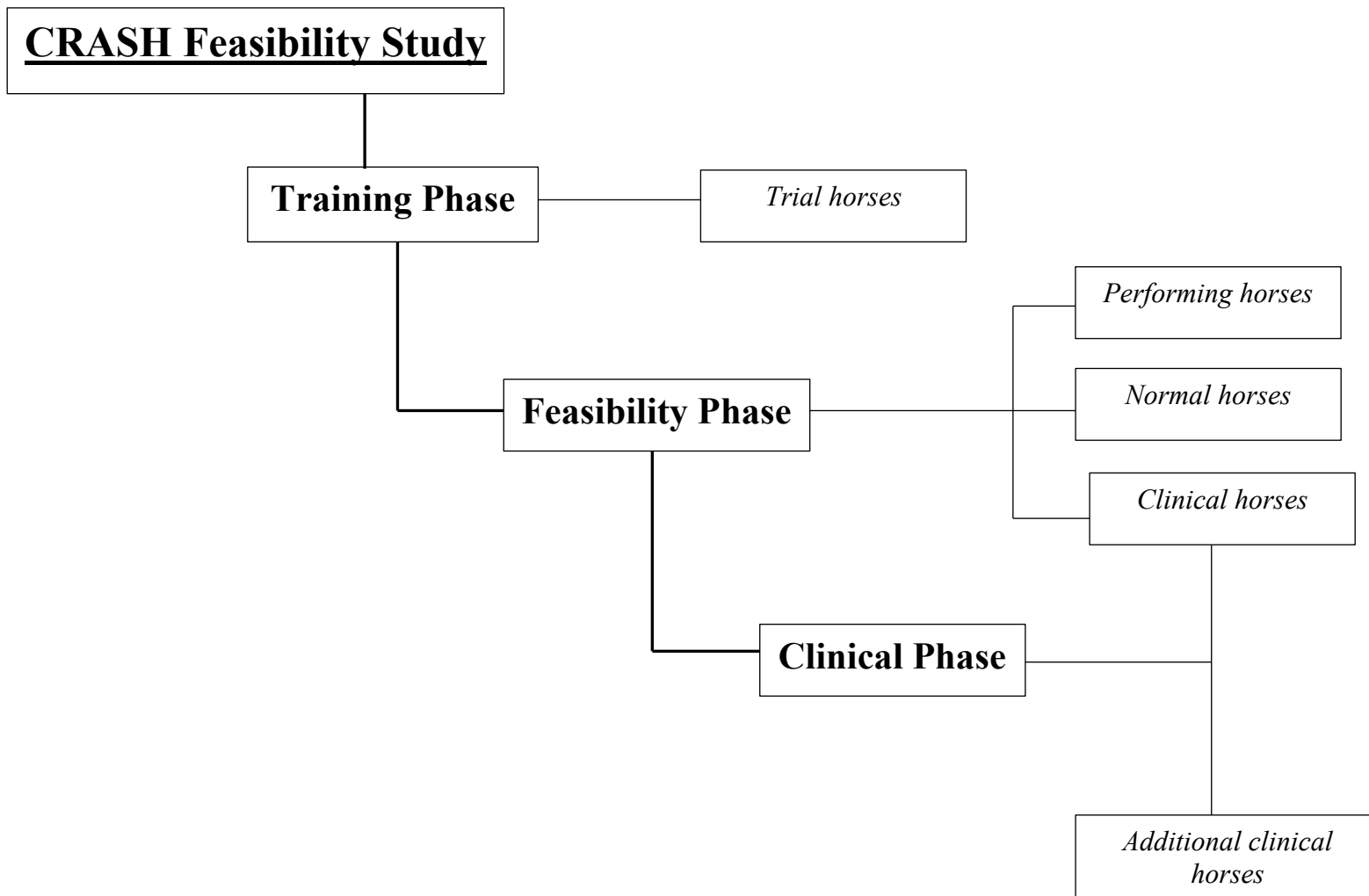

Supplement: Supplementary file 1 — FIGURE S1. CRASH feasibility study [file JVIM-37-1223-s002.pdf]

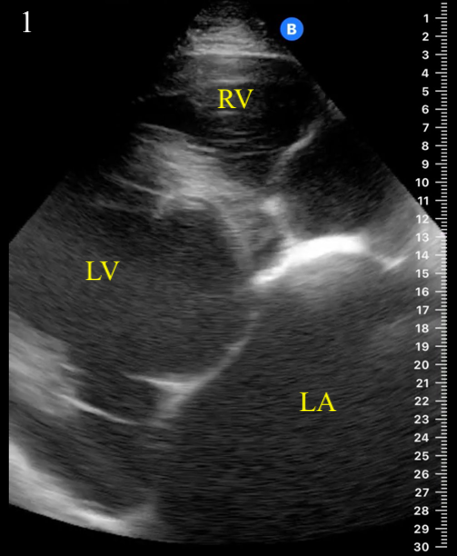

Supplement: Supplementary file 3 — FIGURE S3. Images from horses presented for systemic disease and abnormalities detected in CRASH examination. 1. Right parasternal long axis 4‐chamber view (R4C) ‐ There is severe left atrial enlargement. The left atrium is rounded, several fold larger than the right atrium and expanded beyond the far field of a 30 cm displayed depth. Left Ventricle = LV, Left atrium = LA, Right ventricle = RV, Right atrium = RA. 2. Right parasternal long axis 4‐chamber view (R4C) ‐ The myocardium of the left ventricle is subjectively thickened (star) with an appearance of hypertrophy/pseudohypertrophy. Left Ventricle = LV, Left atrium = LA, Right ventricle = RV, Right atrium = RA. 3. Right parasternal long axis view of the left ventricular outflow tract (LVOT) ‐ The pulmonary artery is larger than the aorta and the interventricular septum is flattened convex right to left suggesting pulmonary hypertension. Aorta = Ao, Pulmonary artery = PA. Left Ventricle = LV, Right ventricle = RV, Interventricular Septum = IVS. 4. Right parasternal long axis view of the left ventricular outflow tract (LVOT) ‐ The aorta has a large nodular lesion (arrow). Aorta = Ao, Pulmonary artery = PA. Left Ventricle = LV, Right ventricle = RV. 5. Right parasternal short axis view at the level of the chordal attachments (SAch) ‐ The myocardium of the left ventricle is subjectively thickened (double arrow) with an appearance of hypertrophy/pseudohypertrophy. Left Ventricle = LV, Right ventricle = RV. 6. Right parasternal short axis view at the level of the aorta (SAAo) ‐ The size of the LA is disproportionally large in comparison with the size of the Ao. Aorta = Ao, Left Atrium = LA, Right ventricle = RV, Pulmonary artery = PA 7. Caudodorsal (CAD) thoracic window (right and left) ‐ A hyperchoic echo of lung with coalescing B lines/comet tail artifacts is seen. 8. Caudoventral thoracic (CAV) window (right and left) ‐ There is a large amount of pleural fluid. Pleural effusion = PE, Diaphragm = Di. [file JVIM-37-1223-s004.zip › JVIM_16674_Supp Fig 3.1.png]

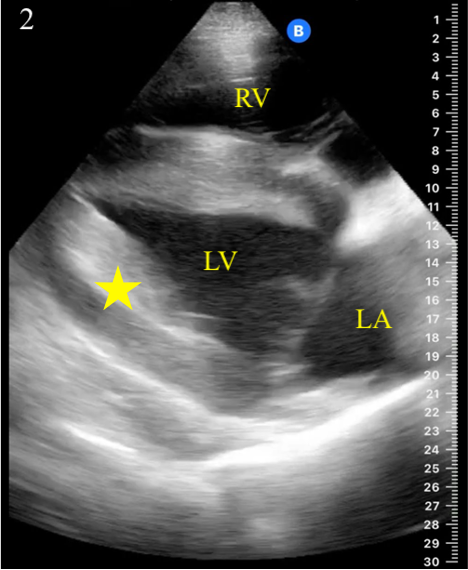

Supplement: Supplementary file 3 — FIGURE S3. Images from horses presented for systemic disease and abnormalities detected in CRASH examination. 1. Right parasternal long axis 4‐chamber view (R4C) ‐ There is severe left atrial enlargement. The left atrium is rounded, several fold larger than the right atrium and expanded beyond the far field of a 30 cm displayed depth. Left Ventricle = LV, Left atrium = LA, Right ventricle = RV, Right atrium = RA. 2. Right parasternal long axis 4‐chamber view (R4C) ‐ The myocardium of the left ventricle is subjectively thickened (star) with an appearance of hypertrophy/pseudohypertrophy. Left Ventricle = LV, Left atrium = LA, Right ventricle = RV, Right atrium = RA. 3. Right parasternal long axis view of the left ventricular outflow tract (LVOT) ‐ The pulmonary artery is larger than the aorta and the interventricular septum is flattened convex right to left suggesting pulmonary hypertension. Aorta = Ao, Pulmonary artery = PA. Left Ventricle = LV, Right ventricle = RV, Interventricular Septum = IVS. 4. Right parasternal long axis view of the left ventricular outflow tract (LVOT) ‐ The aorta has a large nodular lesion (arrow). Aorta = Ao, Pulmonary artery = PA. Left Ventricle = LV, Right ventricle = RV. 5. Right parasternal short axis view at the level of the chordal attachments (SAch) ‐ The myocardium of the left ventricle is subjectively thickened (double arrow) with an appearance of hypertrophy/pseudohypertrophy. Left Ventricle = LV, Right ventricle = RV. 6. Right parasternal short axis view at the level of the aorta (SAAo) ‐ The size of the LA is disproportionally large in comparison with the size of the Ao. Aorta = Ao, Left Atrium = LA, Right ventricle = RV, Pulmonary artery = PA 7. Caudodorsal (CAD) thoracic window (right and left) ‐ A hyperchoic echo of lung with coalescing B lines/comet tail artifacts is seen. 8. Caudoventral thoracic (CAV) window (right and left) ‐ There is a large amount of pleural fluid. Pleural effusion = PE, Diaphragm = Di. [file JVIM-37-1223-s004.zip › JVIM_16674_Supp Fig 3.2.png]

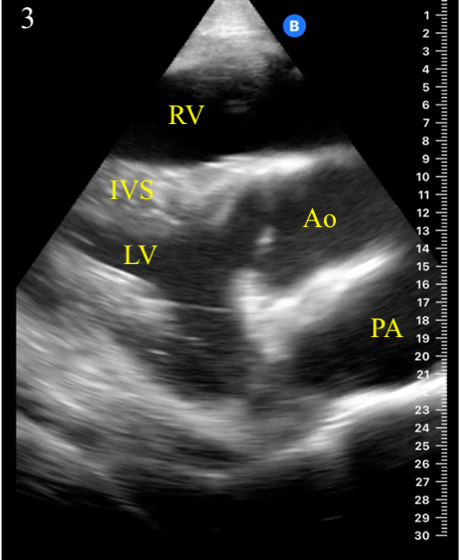

Supplement: Supplementary file 3 — FIGURE S3. Images from horses presented for systemic disease and abnormalities detected in CRASH examination. 1. Right parasternal long axis 4‐chamber view (R4C) ‐ There is severe left atrial enlargement. The left atrium is rounded, several fold larger than the right atrium and expanded beyond the far field of a 30 cm displayed depth. Left Ventricle = LV, Left atrium = LA, Right ventricle = RV, Right atrium = RA. 2. Right parasternal long axis 4‐chamber view (R4C) ‐ The myocardium of the left ventricle is subjectively thickened (star) with an appearance of hypertrophy/pseudohypertrophy. Left Ventricle = LV, Left atrium = LA, Right ventricle = RV, Right atrium = RA. 3. Right parasternal long axis view of the left ventricular outflow tract (LVOT) ‐ The pulmonary artery is larger than the aorta and the interventricular septum is flattened convex right to left suggesting pulmonary hypertension. Aorta = Ao, Pulmonary artery = PA. Left Ventricle = LV, Right ventricle = RV, Interventricular Septum = IVS. 4. Right parasternal long axis view of the left ventricular outflow tract (LVOT) ‐ The aorta has a large nodular lesion (arrow). Aorta = Ao, Pulmonary artery = PA. Left Ventricle = LV, Right ventricle = RV. 5. Right parasternal short axis view at the level of the chordal attachments (SAch) ‐ The myocardium of the left ventricle is subjectively thickened (double arrow) with an appearance of hypertrophy/pseudohypertrophy. Left Ventricle = LV, Right ventricle = RV. 6. Right parasternal short axis view at the level of the aorta (SAAo) ‐ The size of the LA is disproportionally large in comparison with the size of the Ao. Aorta = Ao, Left Atrium = LA, Right ventricle = RV, Pulmonary artery = PA 7. Caudodorsal (CAD) thoracic window (right and left) ‐ A hyperchoic echo of lung with coalescing B lines/comet tail artifacts is seen. 8. Caudoventral thoracic (CAV) window (right and left) ‐ There is a large amount of pleural fluid. Pleural effusion = PE, Diaphragm = Di. [file JVIM-37-1223-s004.zip › JVIM_16674_Supp Fig 3.3.png]

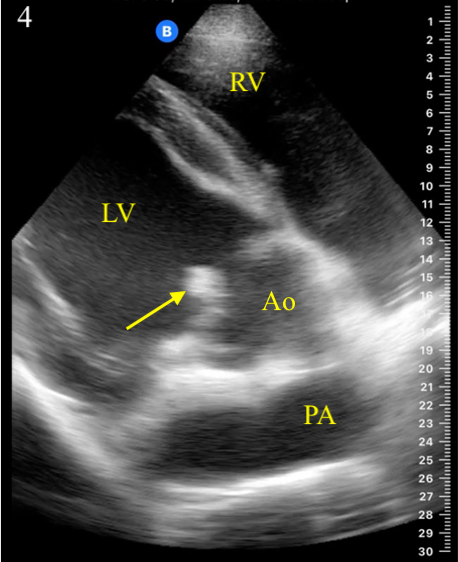

Supplement: Supplementary file 3 — FIGURE S3. Images from horses presented for systemic disease and abnormalities detected in CRASH examination. 1. Right parasternal long axis 4‐chamber view (R4C) ‐ There is severe left atrial enlargement. The left atrium is rounded, several fold larger than the right atrium and expanded beyond the far field of a 30 cm displayed depth. Left Ventricle = LV, Left atrium = LA, Right ventricle = RV, Right atrium = RA. 2. Right parasternal long axis 4‐chamber view (R4C) ‐ The myocardium of the left ventricle is subjectively thickened (star) with an appearance of hypertrophy/pseudohypertrophy. Left Ventricle = LV, Left atrium = LA, Right ventricle = RV, Right atrium = RA. 3. Right parasternal long axis view of the left ventricular outflow tract (LVOT) ‐ The pulmonary artery is larger than the aorta and the interventricular septum is flattened convex right to left suggesting pulmonary hypertension. Aorta = Ao, Pulmonary artery = PA. Left Ventricle = LV, Right ventricle = RV, Interventricular Septum = IVS. 4. Right parasternal long axis view of the left ventricular outflow tract (LVOT) ‐ The aorta has a large nodular lesion (arrow). Aorta = Ao, Pulmonary artery = PA. Left Ventricle = LV, Right ventricle = RV. 5. Right parasternal short axis view at the level of the chordal attachments (SAch) ‐ The myocardium of the left ventricle is subjectively thickened (double arrow) with an appearance of hypertrophy/pseudohypertrophy. Left Ventricle = LV, Right ventricle = RV. 6. Right parasternal short axis view at the level of the aorta (SAAo) ‐ The size of the LA is disproportionally large in comparison with the size of the Ao. Aorta = Ao, Left Atrium = LA, Right ventricle = RV, Pulmonary artery = PA 7. Caudodorsal (CAD) thoracic window (right and left) ‐ A hyperchoic echo of lung with coalescing B lines/comet tail artifacts is seen. 8. Caudoventral thoracic (CAV) window (right and left) ‐ There is a large amount of pleural fluid. Pleural effusion = PE, Diaphragm = Di. [file JVIM-37-1223-s004.zip › JVIM_16674_Supp Fig 3.4.png]

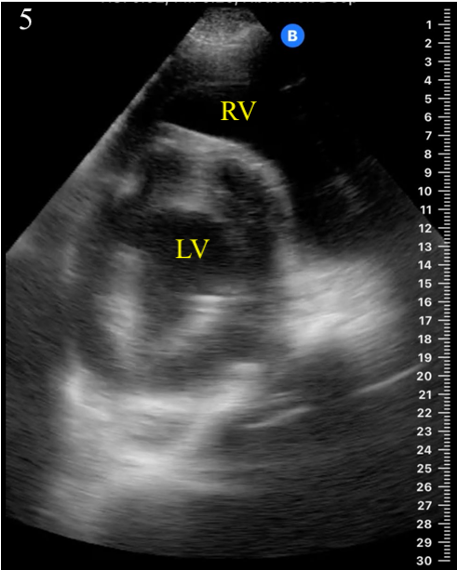

Supplement: Supplementary file 3 — FIGURE S3. Images from horses presented for systemic disease and abnormalities detected in CRASH examination. 1. Right parasternal long axis 4‐chamber view (R4C) ‐ There is severe left atrial enlargement. The left atrium is rounded, several fold larger than the right atrium and expanded beyond the far field of a 30 cm displayed depth. Left Ventricle = LV, Left atrium = LA, Right ventricle = RV, Right atrium = RA. 2. Right parasternal long axis 4‐chamber view (R4C) ‐ The myocardium of the left ventricle is subjectively thickened (star) with an appearance of hypertrophy/pseudohypertrophy. Left Ventricle = LV, Left atrium = LA, Right ventricle = RV, Right atrium = RA. 3. Right parasternal long axis view of the left ventricular outflow tract (LVOT) ‐ The pulmonary artery is larger than the aorta and the interventricular septum is flattened convex right to left suggesting pulmonary hypertension. Aorta = Ao, Pulmonary artery = PA. Left Ventricle = LV, Right ventricle = RV, Interventricular Septum = IVS. 4. Right parasternal long axis view of the left ventricular outflow tract (LVOT) ‐ The aorta has a large nodular lesion (arrow). Aorta = Ao, Pulmonary artery = PA. Left Ventricle = LV, Right ventricle = RV. 5. Right parasternal short axis view at the level of the chordal attachments (SAch) ‐ The myocardium of the left ventricle is subjectively thickened (double arrow) with an appearance of hypertrophy/pseudohypertrophy. Left Ventricle = LV, Right ventricle = RV. 6. Right parasternal short axis view at the level of the aorta (SAAo) ‐ The size of the LA is disproportionally large in comparison with the size of the Ao. Aorta = Ao, Left Atrium = LA, Right ventricle = RV, Pulmonary artery = PA 7. Caudodorsal (CAD) thoracic window (right and left) ‐ A hyperchoic echo of lung with coalescing B lines/comet tail artifacts is seen. 8. Caudoventral thoracic (CAV) window (right and left) ‐ There is a large amount of pleural fluid. Pleural effusion = PE, Diaphragm = Di. [file JVIM-37-1223-s004.zip › JVIM_16674_Supp Fig 3.5.png]

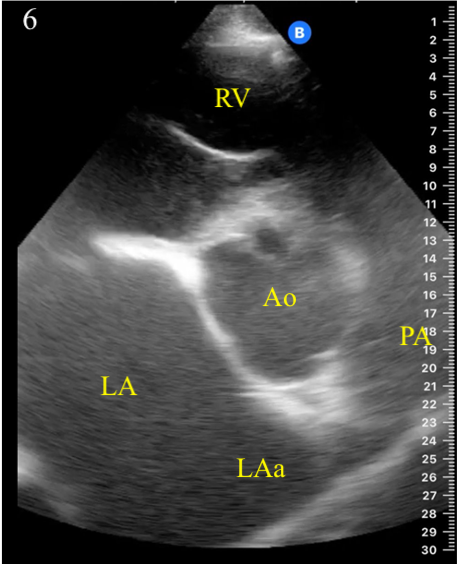

Supplement: Supplementary file 3 — FIGURE S3. Images from horses presented for systemic disease and abnormalities detected in CRASH examination. 1. Right parasternal long axis 4‐chamber view (R4C) ‐ There is severe left atrial enlargement. The left atrium is rounded, several fold larger than the right atrium and expanded beyond the far field of a 30 cm displayed depth. Left Ventricle = LV, Left atrium = LA, Right ventricle = RV, Right atrium = RA. 2. Right parasternal long axis 4‐chamber view (R4C) ‐ The myocardium of the left ventricle is subjectively thickened (star) with an appearance of hypertrophy/pseudohypertrophy. Left Ventricle = LV, Left atrium = LA, Right ventricle = RV, Right atrium = RA. 3. Right parasternal long axis view of the left ventricular outflow tract (LVOT) ‐ The pulmonary artery is larger than the aorta and the interventricular septum is flattened convex right to left suggesting pulmonary hypertension. Aorta = Ao, Pulmonary artery = PA. Left Ventricle = LV, Right ventricle = RV, Interventricular Septum = IVS. 4. Right parasternal long axis view of the left ventricular outflow tract (LVOT) ‐ The aorta has a large nodular lesion (arrow). Aorta = Ao, Pulmonary artery = PA. Left Ventricle = LV, Right ventricle = RV. 5. Right parasternal short axis view at the level of the chordal attachments (SAch) ‐ The myocardium of the left ventricle is subjectively thickened (double arrow) with an appearance of hypertrophy/pseudohypertrophy. Left Ventricle = LV, Right ventricle = RV. 6. Right parasternal short axis view at the level of the aorta (SAAo) ‐ The size of the LA is disproportionally large in comparison with the size of the Ao. Aorta = Ao, Left Atrium = LA, Right ventricle = RV, Pulmonary artery = PA 7. Caudodorsal (CAD) thoracic window (right and left) ‐ A hyperchoic echo of lung with coalescing B lines/comet tail artifacts is seen. 8. Caudoventral thoracic (CAV) window (right and left) ‐ There is a large amount of pleural fluid. Pleural effusion = PE, Diaphragm = Di. [file JVIM-37-1223-s004.zip › JVIM_16674_Supp Fig 3.6.png]

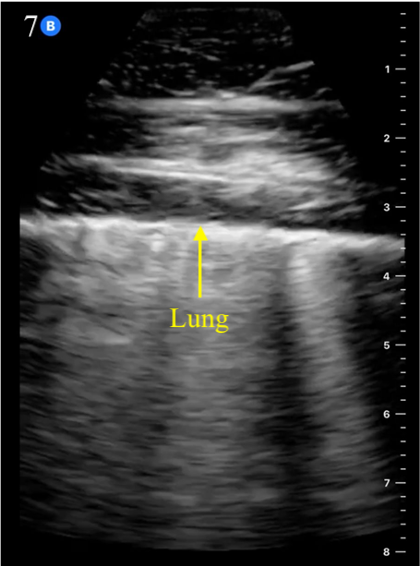

Supplement: Supplementary file 3 — FIGURE S3. Images from horses presented for systemic disease and abnormalities detected in CRASH examination. 1. Right parasternal long axis 4‐chamber view (R4C) ‐ There is severe left atrial enlargement. The left atrium is rounded, several fold larger than the right atrium and expanded beyond the far field of a 30 cm displayed depth. Left Ventricle = LV, Left atrium = LA, Right ventricle = RV, Right atrium = RA. 2. Right parasternal long axis 4‐chamber view (R4C) ‐ The myocardium of the left ventricle is subjectively thickened (star) with an appearance of hypertrophy/pseudohypertrophy. Left Ventricle = LV, Left atrium = LA, Right ventricle = RV, Right atrium = RA. 3. Right parasternal long axis view of the left ventricular outflow tract (LVOT) ‐ The pulmonary artery is larger than the aorta and the interventricular septum is flattened convex right to left suggesting pulmonary hypertension. Aorta = Ao, Pulmonary artery = PA. Left Ventricle = LV, Right ventricle = RV, Interventricular Septum = IVS. 4. Right parasternal long axis view of the left ventricular outflow tract (LVOT) ‐ The aorta has a large nodular lesion (arrow). Aorta = Ao, Pulmonary artery = PA. Left Ventricle = LV, Right ventricle = RV. 5. Right parasternal short axis view at the level of the chordal attachments (SAch) ‐ The myocardium of the left ventricle is subjectively thickened (double arrow) with an appearance of hypertrophy/pseudohypertrophy. Left Ventricle = LV, Right ventricle = RV. 6. Right parasternal short axis view at the level of the aorta (SAAo) ‐ The size of the LA is disproportionally large in comparison with the size of the Ao. Aorta = Ao, Left Atrium = LA, Right ventricle = RV, Pulmonary artery = PA 7. Caudodorsal (CAD) thoracic window (right and left) ‐ A hyperchoic echo of lung with coalescing B lines/comet tail artifacts is seen. 8. Caudoventral thoracic (CAV) window (right and left) ‐ There is a large amount of pleural fluid. Pleural effusion = PE, Diaphragm = Di. [file JVIM-37-1223-s004.zip › JVIM_16674_Supp Fig 3.7.png]

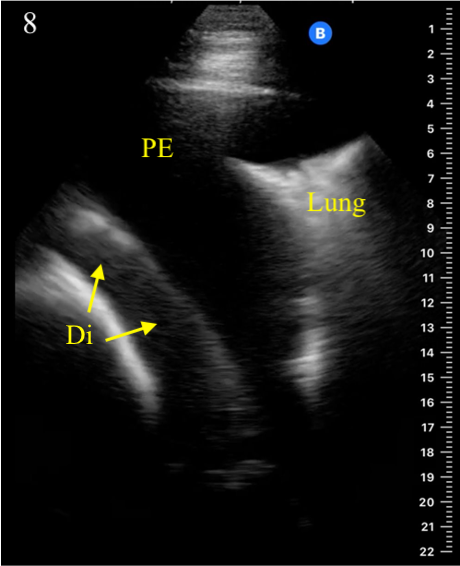

Supplement: Supplementary file 3 — FIGURE S3. Images from horses presented for systemic disease and abnormalities detected in CRASH examination. 1. Right parasternal long axis 4‐chamber view (R4C) ‐ There is severe left atrial enlargement. The left atrium is rounded, several fold larger than the right atrium and expanded beyond the far field of a 30 cm displayed depth. Left Ventricle = LV, Left atrium = LA, Right ventricle = RV, Right atrium = RA. 2. Right parasternal long axis 4‐chamber view (R4C) ‐ The myocardium of the left ventricle is subjectively thickened (star) with an appearance of hypertrophy/pseudohypertrophy. Left Ventricle = LV, Left atrium = LA, Right ventricle = RV, Right atrium = RA. 3. Right parasternal long axis view of the left ventricular outflow tract (LVOT) ‐ The pulmonary artery is larger than the aorta and the interventricular septum is flattened convex right to left suggesting pulmonary hypertension. Aorta = Ao, Pulmonary artery = PA. Left Ventricle = LV, Right ventricle = RV, Interventricular Septum = IVS. 4. Right parasternal long axis view of the left ventricular outflow tract (LVOT) ‐ The aorta has a large nodular lesion (arrow). Aorta = Ao, Pulmonary artery = PA. Left Ventricle = LV, Right ventricle = RV. 5. Right parasternal short axis view at the level of the chordal attachments (SAch) ‐ The myocardium of the left ventricle is subjectively thickened (double arrow) with an appearance of hypertrophy/pseudohypertrophy. Left Ventricle = LV, Right ventricle = RV. 6. Right parasternal short axis view at the level of the aorta (SAAo) ‐ The size of the LA is disproportionally large in comparison with the size of the Ao. Aorta = Ao, Left Atrium = LA, Right ventricle = RV, Pulmonary artery = PA 7. Caudodorsal (CAD) thoracic window (right and left) ‐ A hyperchoic echo of lung with coalescing B lines/comet tail artifacts is seen. 8. Caudoventral thoracic (CAV) window (right and left) ‐ There is a large amount of pleural fluid. Pleural effusion = PE, Diaphragm = Di. [file JVIM-37-1223-s004.zip › JVIM_16674_Supp Fig 3.8.png]
