# Supplementary material for: Feasibility of a point‐of‐care ultrasound protocol for cardiorespiratory evaluation of horses in different clinical settings
Source: J Vet Intern Med. 2023 Mar 28;37(3):1223–32. doi: 10.1111/jvim.16674 (PMC10229348; doi:10.1111/jvim.16674)
Supplement: Supplementary file 2 — FIGURE S2. Body condition scores (range, 1‐9) for horses in feasibility phase of study. [file JVIM-37-1223-s003.pdf]

Supplemental Figure 2. Body condition scores (range, 1 to 9) for horses in feasibility phase of study.[42]

Group	Body Condition Score					
	2	3	4	5	6	7
Normal	0	0	9	11	5	2
Hospitalized	1	3	19	14	7	6
Athletic	0	0	3	11	0	0
