# Supplementary material for: Feasibility of a point‐of‐care ultrasound protocol for cardiorespiratory evaluation of horses in different clinical settings
Source: J Vet Intern Med. 2023 Mar 28;37(3):1223–32. doi: 10.1111/jvim.16674 (PMC10229348; doi:10.1111/jvim.16674)
Supplement: Supplementary file 4 — FIGURE S4. Abnormalities detected with CRASH and the clinical finding(s) that prompted CRASH evaluation for Clinical Phase. Number of cases that had abnormality listed in left column (n=) along with abnormality. Some cases had more than 1 clinical finding that met criteria for enrollment in the clinical phase. [file JVIM-37-1223-s001.pdf]

	Arrhythmia	Tachycardia	Murmur	Increased respiratory effort/rate	Cough	Fever	Colic
Pleural fluid (n = 15)	0	1	0	7	0	8	0
Pericardial fluid (n = 1)	0	0	0	0	0	1	1
Lung pathology (n = 24)	2	1	5	5	5	11	4
Pneumothorax (n = 2)	0	0	0	0	1	2	0
Pulmonary hypertension (n = 7)	1	0	1	3	0	2	0
Left heart disease (n = 14)	5	0	14	0	0	0	0
Valvular or myocardial disease (n = 3)	1	0	2	0	0	1	0
Hypertrophy or pseudohypertrophy (n = 8)	0	2	0	0	1	2	4

Supplemental Figure 4: Abnormalities detected with CRASH and the clinical finding(s) that prompted CRASH evaluation for Clinical Phase. Number of cases that had abnormality listed in left column (n=) along with abnormality. Some cases had more than 1 clinical finding that met criteria for enrollment in the clinical phase.
